# PanCancer analysis of somatic mutations in repetitive regions reveals recurrent mutations in snRNA U2

**DOI:** 10.1038/s41525-022-00292-2

**Published:** 2022-03-14

**Authors:** Pablo Bousquets-Muñoz, Ander Díaz-Navarro, Ferran Nadeu, Ana Sánchez-Pitiot, Sara López-Tamargo, Shimin Shuai, Milagros Balbín, Jose M. C. Tubio, Sílvia Beà, Jose I. Martin-Subero, Ana Gutiérrez-Fernández, Lincoln D. Stein, Elías Campo, Xose S. Puente

**Affiliations:** 1 Departamento de Bioquímica y Biología Molecular, Instituto Universitario de Oncología (IUOPA), Universidad de Oviedo, Oviedo, Spain; 2 Institut d’Investigacions Biomèdiques August Pi i Sunyer (IDIBAPS), Barcelona, Spain; 3 Centro de Investigación Biomédica en Red de Cáncer (CIBERONC), Madrid, Spain; 4 Laboratorio de Oncología Molecular, Laboratorio de Medicina, Instituto Universitario de Oncología (IUOPA), Hospital Universitario Central de Asturias, Oviedo, Spain; 5 Department of Human Cell Biology and Genetics, School of Medicine, Southern University of Science and Technology, Shenzhen, China; 6 Genomes and Disease, Centre for Research in Molecular Medicine and Chronic Diseases (CIMUS), Universidade de Santiago de Compostela, Santiago de Compostela, Spain; 7 Hospital Clinic de Barcelona, Universitat de Barcelona, Barcelona, Spain; 8 Institució Catalana de Recerca i Estudis Avançats (ICREA), Barcelona, Spain; 9 Department of Molecular Genetics, University of Toronto, Toronto, ON Canada; 10 Computational Biology Program, Ontario Institute for Cancer Research, Toronto, ON Canada

**Keywords:** Molecular medicine, Cancer genomics, Data mining

## Abstract

Current somatic mutation callers are biased against repetitive regions, preventing the identification of potential driver alterations in these loci. We developed a mutation caller for repetitive regions, and applied it to study repetitive non protein-coding genes in more than 2200 whole-genome cases. We identified a recurrent mutation at position c.28 in the gene encoding the snRNA *U2*. This mutation is present in B-cell derived tumors, as well as in prostate and pancreatic cancer, suggesting *U2* c.28 constitutes a driver candidate associated with worse prognosis. We showed that the GRCh37 reference genome is incomplete, lacking the *U2* cluster in chromosome 17, preventing the identification of mutations in this gene. Furthermore, the 5′-flanking region of *WDR74*, previously described as frequently mutated in cancer, constitutes a functional copy of *U2*. These data reinforce the relevance of non-coding mutations in cancer, and highlight current challenges of cancer genomic research in characterizing mutations affecting repetitive genes.

## Introduction

The development of Next Generation Sequencing (NGS) technologies has allowed the study of human variation at an unprecedented resolution. In the case of cancer, the completion of different large cancer genomic studies, such as the International Cancer Genome Consortium (ICGC) or The Cancer Genome Atlas (TCGA), has transformed our knowledge of many of these pathologies with the identification of many driver mutations affecting genes previously unsuspected of being mutated in cancer^1–3^. These findings facilitate the development of novel diagnostic approaches, better patient stratification, and the opportunity to develop novel therapeutic strategies.

However, the short read lengths produced by NGS technologies used in these projects hamper the study of mutations in repetitive regions of the genome. In this regard, due to the high sequence identity between these loci, short reads derived from a repetitive region are ambiguously aligned to one of the repeats, not to the one they came from. These reads are assigned a very low mapping quality to reflect the low confidence in the alignment^4–6^. Most somatic mutation callers discard those reads due to their poor mapping quality, preventing the identification of somatic mutations in these regions. In a more complex scenario, some of those regions might not even be present in the reference genome used for these projects (mostly GRCh37), preventing their effective analysis.

Although most protein-coding genes are located in non-repetitive regions, many non-protein coding genes are repetitive, a fact that might have prevented the identification of mutations affecting these functional genes. A clear example is the gene encoding *U1*, a small nuclear RNA (snRNA) that is part of the spliceosome. The human genome contains at least seven identical copies of this gene, as well as dozens of pseudogenes, greatly hindering its analysis^7,8^. However, it has been recently shown that this gene is frequently mutated in different cancer types, with a recurrent mutation on position 3 in more than 50% of Shh medulloblastomas, as well as in different hematological neoplasias and other tumors^9,10^. The identification of highly recurrent mutations in genes located in repetitive regions suggests that these complex regions might contain additional driver mutations previously missed by current analytical pipelines.

## Results

### Development of a computational pipeline to identify somatic mutations in repetitive regions

**Fig. 1:**
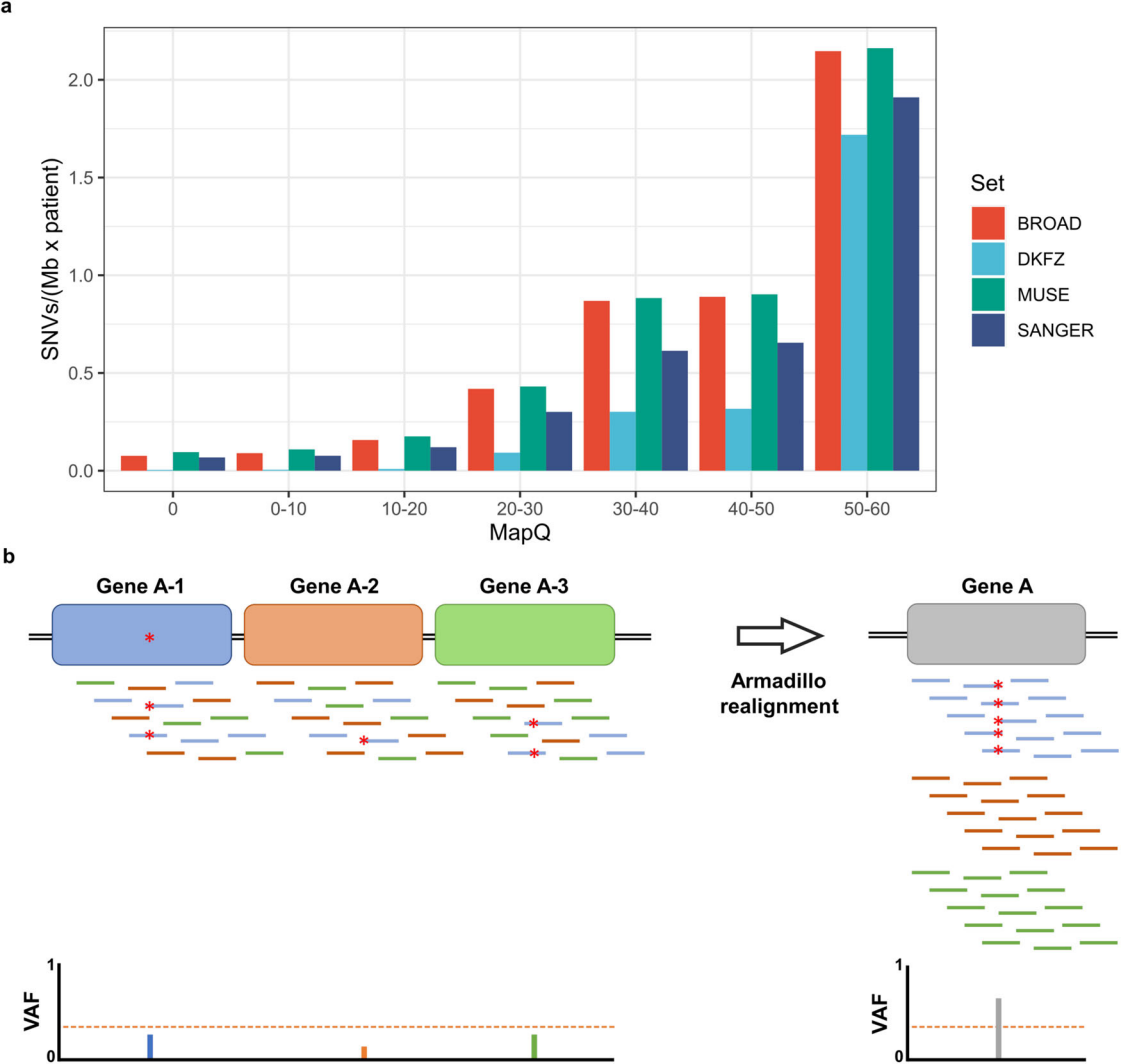
Effect of mapping quality on the identification of somatic mutations. **a** Histogram showing the somatic mutation density of SNVs detected by four different mutation callers on PCAWG data according to the average mapping quality of the reads covering the mutant base. **b** Effect of repetitive copies of a gene on the detection of somatic mutations. Mutated reads are effectively diluted along different copies, reducing the variant allele frequency (VAF) and preventing the identification of somatic mutations. Armadillo aligns all reads derived from repetitive copies to a single reference copy, allowing the reach of a minimum threshold to call a somatic mutation.

To explore the potential relationship between the ability to detect somatic mutations in tumor samples and the repetitive nature of different loci, we used the somatic mutations identified by the ICGC PanCancer Analysis of Whole Genomes (PCAWG) in 2658 tumor samples^2^, and compared the density of mutations identified by different mutation callers at different mapping qualities (Fig. 1a). We observed that the density of somatic mutations per Megabase detected diminished as the mapping quality was lower (an indication of repetitiveness), with almost no mutations detected at loci with a mapping quality of 0, and a 90% reduction in the density of mutations at loci with mapping quality below 20. As the accumulation of somatic mutations is considered to be a stochastic process^11,12^, the absence of mutations in repetitive regions suggests that this fact is likely due to technical limitations and not to a lower incidence of mutations in these regions. Thus, these results show that despite major advances in somatic mutation calling^13–15^, there is a bias in the analysis of somatic mutations using short reads, potentially leading to the loss of bona fide driver mutations located in repetitive regions of the genome. Recent efforts have improved the mutation calling in repetitive regions, but lack enough sensitivity to detect mutations in highly repetitive genes such as U1 snRNA^16^.

Although novel technologies are able to generate longer reads^17,18^, which could solve some of the technical limitations in the analysis of repetitive regions, it is very unlikely that those novel approaches could be applied to the re-analysis of the thousands of cancer genomes sequenced to date, due to both sample availability and technical and economic limitations. Therefore, to study those regions it is necessary to develop analytical pipelines to explore the landscape of somatic mutations in repetitive regions. For this aim, we developed a pipeline, called *Armadillo*, which addresses most of the technical problems associated with the identification of somatic mutations in repetitive regions. Due to the high or even complete sequence identity between some of these regions, when reads containing a mutation are aligned to the reference genome, they could be aligned to different loci, which results in an effective dilution of the mutant reads at any of those positions (Fig. 1b). The fact that the confidence in their alignment is very low, together with the lack of enough mutant reads at any individual locus, prevents the identification of mutations in these regions by most callers. To address this issue, Armadillo takes advantage of the high sequence identity between these loci to create a single reference sequence with only one of those regions. Reads mapped to any of those loci are extracted from the BAM file and aligned to this unique reference sequence. If one copy contained a somatic mutation, all reads containing the mutation will be mapped together (Fig. 1b). Variant calling is performed in tumor- and non-tumor-derived samples, and potential somatic mutations are extracted. Due to the high depth of coverage achieved, sequencing errors accumulated give rise to false positives. To filter out these errors, as most of these repeats are almost identical but usually contain small differences in sequence, potential variants that appear in *cis* with different base changes are discarded, as they likely originated from different loci, and not from a single locus containing a mutation. Finally, to discard germline variants that might be missed due to poor coverage in the non-tumor sample, we used a panel of normal genomes from the ICGC.

### Performance of Armadillo in synthetic and real tumor genome data

Among the different types of genes present in the human genome, a high proportion of non-protein coding genes such as tRNAs, rRNAs, snRNAs and snoRNAs are located in repetitive regions (Supplementary Fig. 1), while less than 13% of exons from protein-coding genes are repetitive. Therefore, we focused our analysis on 342 repetitive regions of interest (RROIs) containing non-protein coding genes (Supplementary Table 1). To determine the performance of Armadillo, we used a set of synthetic genomes containing 2444 mutations randomly distributed across these regions (Supplementary Table 2). The analysis revealed that Armadillo is able to identify 1056 out of 1698 spiked mutations that reached at least three reads supporting the alternative allele (sensitivity 62.2%), with a precision of 99.9% (Supplementary Table 2). The main source of false negatives was due to the trade-off between sequence similarity - to consider two regions as copies—and sensitivity. To recover as many mutations as possible, Armadillo sets loose limits for two sequences being considered as similar. However, some reads from those regions might not align perfectly to the copy used as reference for that repetitive region, resulting in soft clipping. Thus, when mutations are spiked within those regions, it results in the accumulation of many variants within a small region of the read forcing soft clipping and failure to detect the spiked in mutation. The other factor affecting sensitivity was coverage, with a drop at coverages below 20×. In addition, we detected 1 potential somatic mutation not introduced in Bamsurgeon but generated due to subsampling.

To evaluate the performance in real samples we applied Armadillo to 57 mantle cell lymphoma (MCL) cases^19^. We identified 22 somatic mutations (Supplementary Table 3), corresponding to 0.39 per case, resulting in a mutational burden of 1.8 mutations per Mb of repetitive sequence analyzed (0.21 Mb), a 50% higher than the mutational burden reported for MCL (1.2 mutations per Mb)^19^. However, 12 of the 22 mutations were located in the 5′UTR of *WDR74*, a region which had been found mutated in different cancers^3,20^, and where *RNU2-2P* is located. This enrichment in a potential driver gene might be responsible for the apparently higher mutational burden observed in repetitive regions. When those potentially driver mutations were excluded, the observed mutational burden in repetitive regions was 0.8 mutations per Mb, slightly lower than that reported for MCL^19^, and in agreement with the sensitivity observed in synthetic genomes, suggesting that Armadillo is able to detect somatic mutations in real tumor samples.

### PanCancer analysis of somatic mutations in repetitive genes

We then applied Armadillo to 2240 WGS cases from the ICGC consortium’s collaboratory server^2,21^, including most PCAWG samples (Supplementary Table 4). This analysis resulted in the identification of 1084 somatic mutations in repetitive genes (Supplementary Table 5). One of the most frequently mutated genes was snRNA *U1*, mutated in 112 cases. This allowed us to confirm the presence of the previously reported c.3A>C mutation in *U1*^9^ both in CLL (4%) and hepatocellular carcinoma (2.4%), the *U1* c.3A>G mutation in pediatric brain cancer (7.7%) and pediatric medulloblastoma (8%)^10^, as well as *U1* c.7A>G in the malignant lymphoma cohort (3%) (Supplementary Table 5). Only the previously described *U1* c.9C>T mutation^9^ could not be detected due to the fact that one of the copies of *U1* (*RNUV1-2*) has this variation at position 9 and was filtered out in the control-matched samples. These findings support the sensitivity of Armadillo to detect somatic mutations in repetitive regions, an improvement over previous studies of mutations in repetitive genes^16^ that missed the 42 PCAWG cases with a c.3A>C/G mutation in U1 snRNA, despite an overall higher sensitivity on simulated data (data not shown).

We identified mutations in 212 RROIs out of the 342 analyzed (62%) (Supplementary Table 5). Interestingly, three of the 212 RROIs accumulated more than 33% of all mutations. One of those RROIs corresponded to the bona fide driver gene *U1* discussed above, while the other two corresponded to the *U2* snRNA (*RNU2*) and to a 5S rRNA pseudogene (*RNA5SP452*). Other frequently mutated genes included tRNAs for Glu_UUC, Asp_GUC or Leu_CAG, with more than 15 mutations each.

### The *U2* c.28C mutation is frequent in hematological tumors, prostate and pancreatic cancer

**Fig. 2:**
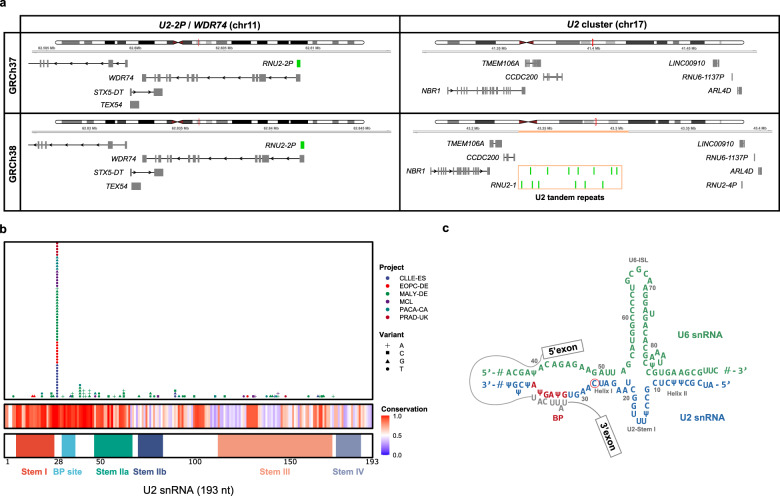
snRNA U2 is recurrently mutated at position c.28. **a** Gene organization of the *WDR74/U2-2P* locus in chromosome 11 and the *U2* cluster locus in chromosome 17 in human reference genome GRCh37 and GRCh38. The *U2* cluster from chromosome 17 is missing in GRCh37 (new sequence in GRCh38 is highlighted in orange), which leads to *U2*-derived reads to align to *U2-2P* in chromosome 11. **b** Schematic representation of *U2* somatic mutations identified by Armadillo in 2.297 WGS samples from PCAWG and MCL. Conservation and structure features were added from RFAM database. **c** Model of U2 secondary structure during splicing. The red circle shows c.28C > T mutation.

The analysis of somatic mutations across 57 MCL samples (Supplementary Table 3) revealed that the gene encoding the *U2* snRNA (*RNU2*), which is part of the spliceosome and involved in the recognition of the branching site during RNA splicing^22^, was the most frequently mutated gene with a total of 12 mutations. Particularly, a mutation affecting base 28 (*U2* c.28C>T) was found recurrently mutated in 5 cases, while the same base was mutated to G (c.28C>G) in one case. *RNU2* is a hypervariable macrosatellite located in chromosome 17q21, consisting of a 6.1 kb unit with 6–82 copies per allele^23,24^, and had not been previously identified mutated in cancer. In fact, the GRCh37/hg19 human reference genome used to align both our MCL cohort as well as ICGC PCAWG samples is incomplete at this locus and does not contain this cluster of *U2* copies, which would make *U2* mutations undetectable (Fig. 2a). However, this reference genome contains a sequence annotated as a pseudogene (*RNU2-2P*) located within the 5′UTR of a transcript of *WDR74* in chromosome 11 (Fig. 2a). This locus has 97% identity to *U2*, so in the absence of the *U2* locus in the reference genome, most of the reads that should align to the *U2* locus on chromosome 17 are aligned to this annotated pseudogene. Indeed, this non-coding region had been previously considered as a potential cancer driver gene with a regional recurrence just below *TERT*^3,20,25^, but no molecular mechanism had been found yet for the accumulation of mutations in the 5’-flanking region of *WDR74*. Our results suggest that *U2* is the gene that accumulates most of these somatic mutations, but due to the lack of the *U2* locus in GRCh37, most reads were mapped to an annotated untranslated exon of *WDR74*.

Despite the incompleteness of the GRCh37 reference genome, the ICGC-PCAWG used as reference sequence hs37d5 (GRCh37 plus decoy)^2^. Interestingly, the decoy contig hs37d5:7,364,539–7,364,724 contains a copy of *U2*. Therefore, for the ICGC-PCAWG genomes, reads coming from the *U2* loci are mapped to the decoy instead of to the 5′UTR of WDR74. This fact allowed us to use the hs37d5-aligned ICGC-PCAWG genomes to extract U2-derived reads that were mapped either to the 5’UTR of *WDR74* or to the decoy region, and apply Armadillo to detect somatic mutations affecting *U2* or the annotated *U2-2P* pseudogene. This analysis resulted in the identification of 213 cases with somatic mutations in U2, including 67 cases harboring a mutation at position c.28 of *U2* or the *U2-2P* (Fig. 2b, c and Supplementary Table 5).

The *U2* c.28C > T mutation was present mostly in hematological and prostate cancer tumors from the PCAWG data (Fig. 2b). We found this recurrent mutation in three hematological tumors: our MCL cohort with a frequency of 8.7% (5/57), the ICGC CLL-ES at 7.3% (11/149), and germinal-center derived B-cell malignant (non-Hodgkin) lymphoma at 9% (9/99). In prostate cancer we identified the c.28C>T mutation in early onset prostate cancer EOPC-DE at a frequency of 4.5% (8/179), and in prostate adenocarcinoma PRAD-UK at 13.8%, although with a low sample size (5/38 cases) (Fig. 2b and Supplementary Table 5). This data confirms previous studies showing that prostate cancer is enriched in mutations affecting the 5′-flanking region of *WDR74*^25^, although our data suggest that *U2* might have been the real target of these mutations.

Interestingly, malignant lymphomas corresponding to the German germinal-center derived B-cell malignant (non-Hodgkin) lymphoma ICGC project (MALY-DE)^26^, showed not only the c.28C>T mutation in 9 cases, but also an alternative change affecting the same position (c.28C>G) in 9 cases, and a c.28C>A substitution in one case (Fig. 2b and Supplementary Table 5). This c.28C>G mutation was also detected in MCL and CLL, with one case each, as well as in pancreatic cancer (PACA-CA), with 5/214 cases. Altogether, 18% of cases from MALY-DE have a mutation at position c.28 in *U2*, including 25% of diffuse large B-cell lymphomas (10/40), 19% of follicular lymphomas (6/31), 100% of small B-cell lymphomas (2/2), and 5% of Burkitt lymphomas (1/17), which along with MCL and CLL, suggests that the recurrent mutation at position c.28 of *U2* might constitute a driver candidate in these tumors.

### Verification of the U2 c.28C>T mutation in primary tumor samples

Due to the repetitive and complex nature of *U2*, we used two orthogonal validation strategies to confirm the presence of somatic mutations in this gene using RNA and genomic DNA. First, we confirmed that in all mutated cases from the CLL-ES and MCL projects for which there were RNA-seq data (*N* = 13), the *U2* c.28C>T mutation was expressed, while it was not detected in RNA-seq data from all non-mutated cases (*N* = 95), confirming that the mutation is expressed (Supplementary Fig. 2). Second, we used rhAmp SNP genotyping PCR to confirm the presence of *U2* c.28C>T mutation in genomic DNA, using two different probes to discriminate between canonical *U2* and the *U2-2P* sequence. We genotyped the CLL cases analyzed by NGS, and extended the analysis up to 823 samples. We identified 28 patients with the c.28C>T mutation in *U2*, while 11 patients had the c.28C>T mutation in *U2-2P* and one has the mutation but could not be definitely assigned to *U2* or *U2-2P* (Supplementary Table 6). In all cases with available WGS data, the NGS findings agreed with the rhAmp results, except three discrepant cases (Supplementary Table 6). One called wild type (WT) by rhAmp while clearly mutated by NGS, and two called mutated by rhAmp but wild type by NGS due to a low number of reads supporting the variant.

### The 5′-flanking region of *WDR74* contains an expressed copy of *U2*

**Fig. 3:**
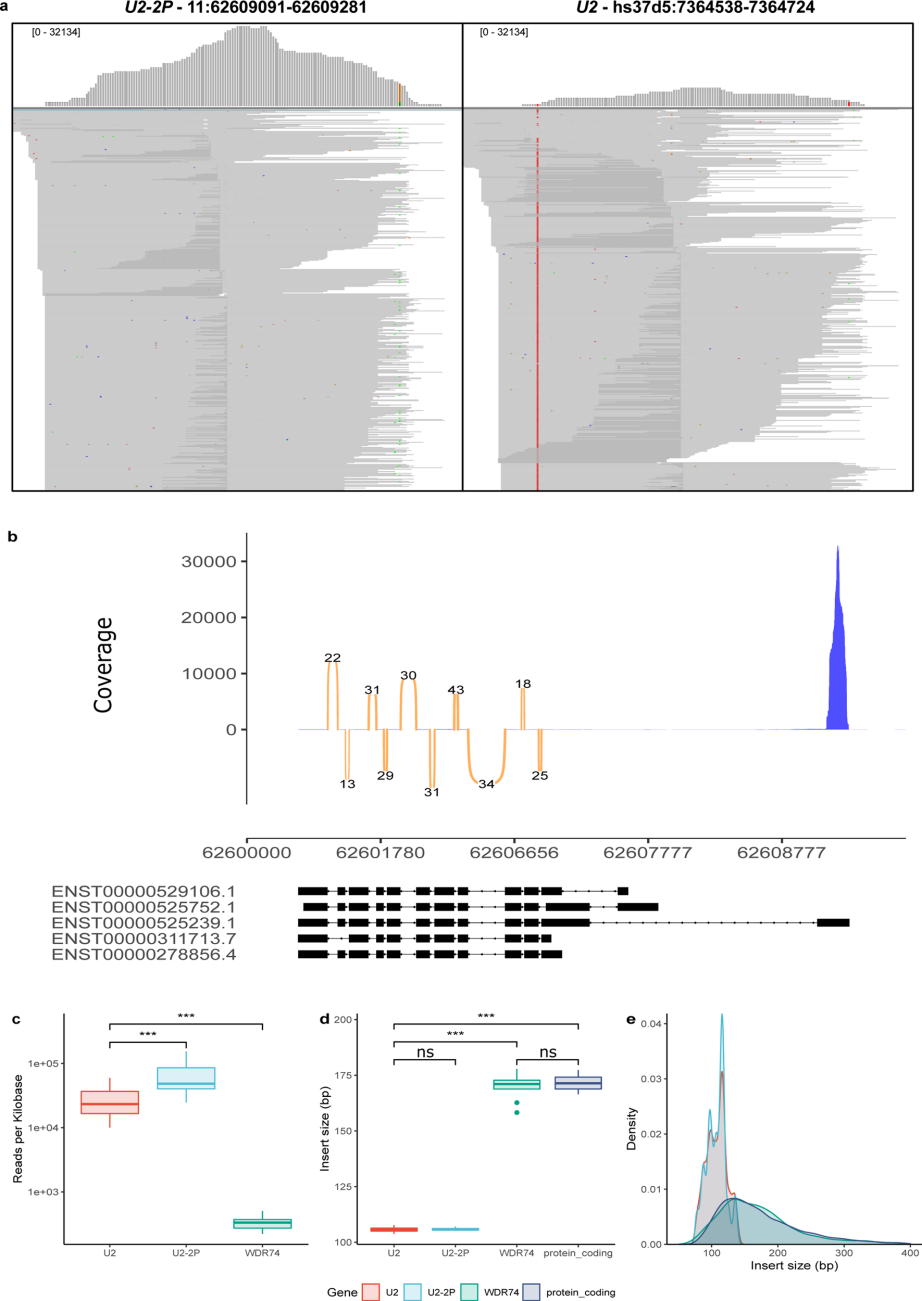
*RNU2-2P* is expressed and is not part of the *WDR74* transcriptional unit. **a** Representative example of RNA-seq from a MCL sample aligned to the GRCh37 decoy reference genome (hs37d5) showing the *U2-2P* locus at chromosome 11, and the *U2* sequence at the decoy region hs37d5:7,364,538-7,364,724. Only read pairs unequivocally derived from *U2* or *U2-2P* based on the different sequence between both copies are shown. Note that the reference decoy sequence has a change with respect to canonical *U2*, resulting in the detection of a variant in all *U2*-derived reads aligned to the decoy sequence. **b** Sashimi plot showing the depth of coverage and splicing events in the *WDR74* locus as well as gene models for *WDR74* derived from Ensembl. Number of reads supporting each splicing event is indicated. For *U2-2P*, only informative reads that could be unequivocally aligned to this locus were represented. **c** *U2-2P* is expressed at higher levels than *U2*, and orders of magnitude higher than *WDR74* (N=12 MCL RNA-seq samples). **d**, **e** Insert size distribution for reads derived from *U2*, *U2-2P*, *WDR74* or protein coding genes. Insert size from *U2-2P*-derived reads are shorter than those aligned to *WDR74* exons or those derived from protein coding genes, and similar to those aligned to the *U2* locus present in the decoy sequence. (**P < 0.01; ***P < 0.001; ns: not significant). Boxplots elements represent: center line = median, upper and lower hinges = 25 and 75% percentiles, upper and lower whisker = mean ± 1.5*IQR.

The analysis of WGS and RNA-seq data, as well as rhAmp results, showed that in some samples the c.28C>T mutation was unequivocally derived from the *U2-2P* locus in the 5′UTR of *WDR74*. The presence of the same recurrent mutation in *U2* as well as in one annotated pseudogene raises questions about the status of *U2-2P* as a pseudogene. In fact, the lack of information about the annotation of *U2-2P* as a pseudogene prompted us to determine whether this locus was a pseudogene or a functional copy of *U2*. According to GTEx^27^, the *WDR74* exon containing *U2-2P* is not included in the transcripts generated in most human tissues with the exception of testis (Supplementary Fig. 3). To determine whether *U2-2P* was expressed in primary tumors, we analyzed RNA-seq data from different MCL samples. This analysis revealed that the *U2-2P*/5′UTR of *WDR74* had a coverage above 30,000 reads per base, while canonical *U2* had more than 15,000 (Fig. 3a–c and Supplementary Fig. 4). By contrast, the depth of coverage for *WDR74* was very homogeneous in exons 2–12, but only at less than 70 reads per base (Fig. 3b, c), strongly suggesting that they correspond to different transcriptional units.

Similar to the data obtained by GTEx, there were no reads supporting a canonical splicing between this annotated first exon of *WDR74/U2-2P* (ENSE00002157686) and the next exons of *WDR74* (ENSE00002179520 or ENSE00002315240) (Fig. 3b), further suggesting that they correspond to different transcripts. An additional line of evidence supporting this hypothesis comes from the analysis of insert sizes from read pairs mapped to *U2-2P*. While reads derived from exons 2–12 of *WDR74* or any coding gene had a broad insert size distribution, with mean 166 bases (Fig. 3d, e), those derived from real *U2* genes had a narrow insert size distribution of about 105 bases due to the small length of the *U2* transcript (193 bases). Interestingly, reads derived from *U2-2P* showed a similar insert size distribution from those derived from *U2*, and very different from those derived from *WDR74* exons 2–12 or other coding genes (Fig. 3d, e), emphasizing the hypothesis that *U2-2P* is a transcribed copy of *U2* located outside the chromosome 17 cluster.

**Fig. 4:**
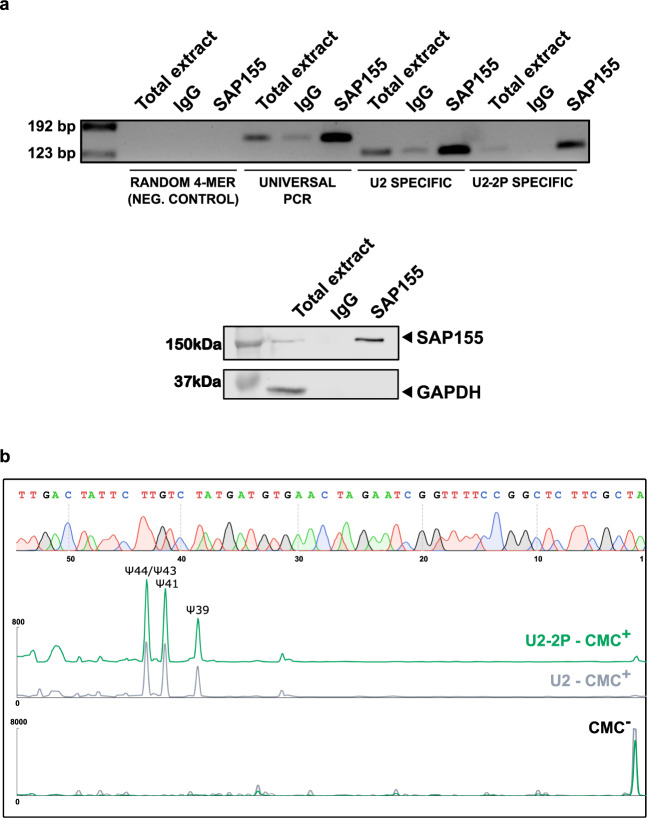
*U2-2P* is incorporated in the spliceosome and post-transcriptionally modified. **a** RT-PCR of *U2* and *U2-2P* with specific primers for each copy or with common primers for both on total extracts from CLL JVM3 cells, or in immunoprecipitated material with anti-SAP155 antibody or with an isotype IgG. In the lower panel, control western blot for the detection of SAP155 in total cell extracts, immunoprecipated with control IgG or with anti-SAP155 antibody from the same experiment as above. **b** Pseudouridine mapping using U2- or U2-2P-specific primers showing similar pseudouridylation patterns in both snRNAs. On top, electropherogram corresponding to the Sanger sequence of *U2/U2-2P* with the same primer used for the pseudouridylation analysis.

*U2-2P* has seven nucleotide changes when compared to the canonical human *U2* sequence, four of the seven substitutions are identical to the canonical mouse or rat *U2* sequences (Supplementary Fig. 5), and the others do not affect any critical region, suggesting that the nucleotide changes might not impair the function of this copy of *U2*. To further determine if *U2-2P* could be a functional gene, we explored whether *U2-2P* was incorporated into the spliceosome and whether it was post-transcriptionally modified. Thus, we immunoprecipitated the U2 spliceosomal complex from JVM3 cells using an antibody against SAP155, and performed reverse transcription with specific oligonucleotides recognizing either *U2* or *U2-2P*. PCR amplification resulted in the detection of both *U2* and *U2-2P* snRNAs in the immunoprecipitated RNA (Fig. 4a), while a control oligonucleotide in which the four specific bases distinguishing *U2* from *U2-2P* were swapped for other bases, did not result in any amplification, confirming that *U2-2P* is incorporated in the spliceosome. Next, taking into consideration that *U2* experiences numerous post-transcriptional modifications, being of particular relevance the extensive conversion of uridines to pseudouridines, we analyzed pseudouridylation in *U2* and *U2-2P* using specific oligonucleotides for each gene. We found that *U2-2P* had a similar pseudouridylation profile as *U2* including the presence of pseudouridylated bases at positions ψ41, ψ43, and ψ44 (Fig. 4b). Altogether, these results strongly suggest that the *U2-2P* locus on chromosome 11, previously annotated as an *U2* pseudogene within the 5′UTR of a rarely used exon of *WDR74*, encodes a functional copy of *U2* subject to the same post-transcriptional modifications and incorporated in the spliceosome.

### Germline *U2* c.28C variants constitute rare events

Although the *U2* c.28 C>T or c.28 C>G changes were clearly detected as somatic mutations in most samples harboring these alterations, with no reads supporting these variants in the germline-matched normal samples, rhAmp analysis detected a few cases in which the c.28C>T or c.28C>G substitutions were already present in germline DNA from the patient (Supplementary Table 6). Reanalysis of 2,297 WGS cases from PCAWG and MCL identified 13c.28C>T and 11c.28C>G germline mutations missed by Armadillo due to the presence of the same variant in the paired normal sample (Supplementary Table 7). To explore the possibility that this change might constitute a polymorphism, we performed genotyping for the c.28C>T mutation using a rhAmp assay in 401 individuals from Spanish origin and with no known cancer-related pathology. We detected one donor with the c.28C>T substitution, resulting in a frequency of 0.25%, comparable to that obtained from the normal samples of PCAWG (0.5%, Fischer test *P* = 0.7). These results discard the hypothesis that this mutation could constitute a common polymorphism in our population, and despite the presence as germline variant in some individuals, it does not explain the high frequency of somatic mutations observed in hematological B-cell tumors and prostate or pancreatic cancer.

### Clinical characteristics of *U2* c.28C-mutated cases

Finally, we explored the association of the *U2* c.28C>T mutation with different clinical parameters in 823 CLL cases for which the *U2/U2-2P* mutational data had been confirmed by rhAmp or WGS, and in 38 prostate cancer cases. A total of 40 CLL cases carried the c.28C>T mutation, 28 of them in *U2*, 11 in *U2-2P* and one could not be determined if in *U2* or *U2-2P*. An additional case had the c.28C>G substitution. In all but one case the mutation was somatic. These data show that the c.28C>T mutation in *U2/U2-2P* is present at a frequency of 4.8%, representing one of the top seven genes most frequently mutated in CLL^28^.

**Fig. 5:**
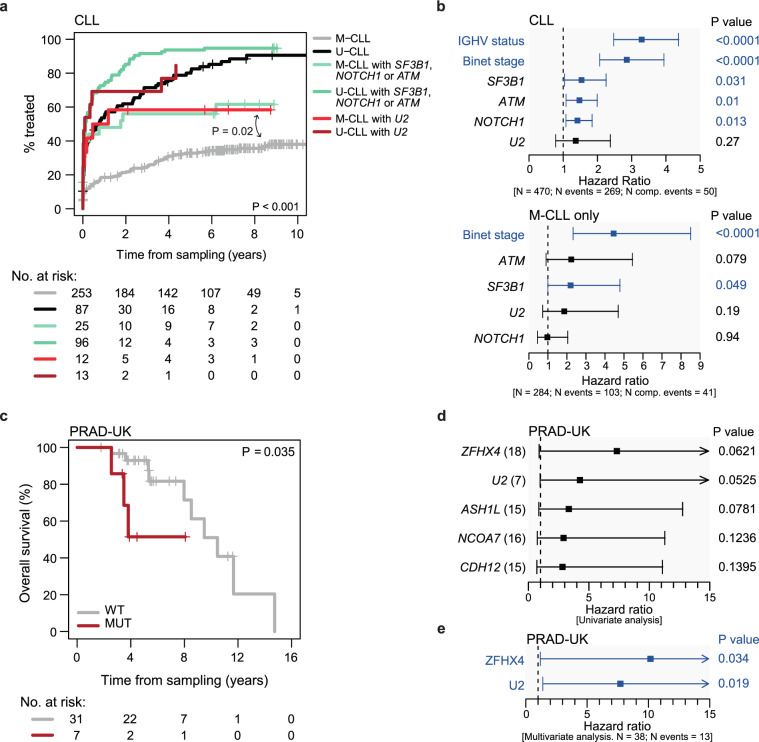
Effect of *U2* c.28 mutation on progression. **a** Univariate Cox regression for *U2* c.28C>T in CLL samples on TTFT. Cases were stratified based on their IGHV status (M-CLL: IHGV mutated; U-CLL: IGHV unmutated) and the presence/absence of high risk mutations in *ATM*, *NOTCH1*, or *SF3B1*. **b** Multivariable Cox regression to evaluate the impact of *U2* c.28C>T mutation on TTFT among known covariates. **c** Kaplan–Meier analysis of prostate adenocarcinoma cases (PRAD-UK) showing the effect of the c.28C > T mutation on overall survival (*P* = 0.035, Univariate Cox regression). **d** Univariable Cox regression with known prostate drivers with clinical impact. **e** Multivariate analysis with *ZFHX4*.

For 594 CLL cases for which molecular and clinical data were available^28,29^ we observed no differences between *U2* c.28C mutated and unmutated patients in terms of time of sampling (pre/post treatment), diagnosis (monoclonal B-cell lymphocytosis/CLL), Binet stage, IGHV mutational status and epigenetic subgroups, although it is slightly more frequent in CLL with unmutated IGHV (U-CLL) than mutated IGHV (M-CLL) cases (Supplementary Fig. 6), the two main subtypes of CLL^30^. The presence of the *U2* mutation appears to be associated to a shorter time to first treatment (TTFT) in univariate analysis, even when cases were stratified based on their IGHV status (Fig. 5a and Supplementary Fig. 6b). Of note, within the M-IGHV subgroup of cases, which is a relatively indolent disease, *U2*-mutated cases had a significantly shorter TTFT than WT cases and similar to that of M-IGHV cases carrying alterations in known driver genes including *NOTCH1*, *ATM* and/or *SF3B1*. In a multivariate model, although not significant (*P* = 0.27), the prognostic value of *U2* (hazard ratio and 95% confidence interval) was similar to that of *ATM* and *SF3B1* mutations (Fig. 5b). We compared the co-occurrence of the *U2/U2-2P* c.28C>T mutation with other previously defined CLL driver alterations (Supplementary Fig. 7). Although no statistically significant associations were found, we observed a high co-occurrence of the *U2* mutation with some driver alterations. Thus, 17% of *U2*-mutated cases had gain of 2p16 vs. only 4% of *U2*-wt cases, while 10% of *U2*-mutated cases had loss of 14q24 vs. 3% of unmutated cases.

In the case of prostate adenocarcinoma (PRAD-UK), *U2* c.28 C>T mutation was detected in 18% of cases (7/38). Univariate cox regression analysis showed that *U2* c.28C>T mutation had a negative impact on overall survival (*P* = 0.035) with a similar impact to other previously described mutated genes^31^ (Fig. 5c and Supplementary Fig. 6d), and independent in a bivariate model including the *ZFHX4* gene, which was the previously known driver gene with the highest prognostic value in this cohort (Fig. 5d). Although larger cohorts are needed, these results suggest that *U2* mutation might be clinically relevant also in prostate adenocarcinoma.

## Discussion

The last decade has been a remarkable period in the field of cancer genomics as NGS technologies have allowed a non-hypothesis driven study of tumor genomes at an unprecedented resolution^2^. These studies have identified novel driver genes, structural variations or mutational signatures which in many cases provided clinical information useful for prognosis, or discovered novel molecular targets that can be used for therapeutic intervention^32,33^. Nonetheless, the ability to detect a somatic mutation relies on the availability of an accurate reference genome to which sequencing reads are aligned, as well as on the development of somatic mutation callers that are able to extract this information from aligned genomes. In this study we confirmed that state of the art somatic mutation callers, such as those used for the analysis of WGS in the PCAWG project^2^, are biased towards unique regions, failing to detect somatic mutations in repetitive regions. This limitation has not been properly addressed due to the fact that the vast majority of repetitive elements in the human genome, such as Alu or LINE1 elements, are not functional, and therefore, their study has not been considered a priority among the plethora of novel driving mutations identified by the analysis of non-repetitive genes. However, some functional genes are highly repetitive, with copies arranged either in cluster or dispersed in different chromosomal loci^34^. The inability of somatic mutation callers to identify mutations in these regions might have prevented the identification of driver genes located within these loci.

In this work, we developed a pipeline for the analysis of somatic mutations in repetitive regions, and applied it to more than 2200 WGS tumor-normal pairs from the PCAWG and MCL projects^2,19^. This analysis showed that the burden of somatic mutations in repetitive regions is similar to that in unique regions, in agreement with the general view that the accumulation of somatic mutations is a stochastic process^11,12^. Similar to genes located in unique regions, while most repetitive genes are mutated, a few of them accumulate a large proportion of these somatic mutations, suggesting that they might represent driver genes such as the previously reported c.3A>C/G in the snRNA U1^9,10^. Armadillo’s performance is not constrained by highly repetitive genes such as GeneticThesaurus^16^, but at the cost of lower sensitivity, suggesting that both methods might be complementary.

Among the recurrently mutated genes, we found that the gene encoding the snRNA U2 (*RNU2*) has a recurrent somatic mutation at position c.28 in 73 tumors from the PCAWG and MCL projects. Similar to U1, the U2 snRNA forms part of the spliceosome, and is responsible for the recognition of the 3′-splicing site by hybridizing with the intron at the branching point region^35^. Protein components of the U2 snRNP such as SF3B1, U2AF1, or U2AF2 have been found frequently mutated in different cancers with a higher incidence in hematological tumors^36–39^. However, analysis of RNA-seq data from primary CLL or MCL tumors or cell lines infected with vectors expressing the *U2* c.28C>T mutation, failed to detect significant differences in gene expression or splicing (data not shown), suggesting that the mechanism by which this mutation exert its effects is not by interfering with intron splicing. The identification of a recurrent somatic mutation in *U2*, with a higher incidence in hematological tumors, suggests that mutations in the snRNA component of the U2 particle could also contribute to tumorigenesis, extending the relevance of this component in cancer.

We found two major mutations in *U2*, c.28C>T and c.28C>G, and those mutations appeared to be tumor-specific. Thus, the *U2* c.28C>T mutation was present mostly in B cell-derived hematological tumors such as CLL and MCL, or in prostate tumors, and the c.28C>G mutation was observed mainly in pancreatic cancer samples, while diffuse large B-cell lymphoma and follicular lymphoma had both mutations at a similar frequency. These results suggest that the *U2* c.28C somatic mutation might constitute a driver event in these specific tumors, at a relatively high frequency (up to 25% of tumors), that was previously missed due to the bias of mutation callers towards unique regions.

Our analysis also revealed another important and usually overlooked factor in the analysis of somatic mutations as is the completeness of the reference genome used for the alignment of sequencing reads^40^. *RNU2* forms part of a hypervariable macrosatellite with 6–82 copies per allele^24^. This locus was first included in the human reference genome in version GRCh38, while it was absent in all previous versions, including those used for large cancer genomic consortiums such as TCGA or ICGC, where GRCh37 continues to be used as reference^2,28^. The absence of *U2* from the reference genome makes almost impossible the identification of somatic mutations in this repetitive gene. However, the presence of an almost identical copy of *U2* in chromosome 11q12 resulted in the finding of recurrent mutations in this locus due to the alignment of *U2*-derived reads to this almost identical region. In fact, previous studies had classified this non-coding element as recurrently mutated in different tumors, with a mutation burden just below *TERT*^3,20,25^. However, these mutations were not detected in more recent projects such as those from the PCAWG, as this project used the GRCh37 reference genome with the inclusion of a decoy sequence^2,3^. While this decoy sequence reduced the number of false positive calls due to misalignment of certain repetitive sequences, the presence of a *U2* copy in the decoy led to all *U2*-derived reads to be aligned to the decoy, effectively suppressing the ability to detect mutations in this gene.

Our results also highlighted another essential aspect in the interpretation of mutations as is the existence of an accurate labeling of functional regions. Since the completion of the human reference genome, a great effort has been made to annotate all functional elements^41,42^, a difficult task due to the absence in most cases of experimental validation data. In the case of *U2*, a copy present in chromosome 11 had been labeled as an *U2* pseudogene (*RNU2-2P*), and the region as the 5’UTR of a *WDR74* transcript, despite the lack of strong evidence in this sense. We showed that *U2-2P* is highly expressed, constitutes a different transcript unit from *WDR74* and experiences similar post-transcriptional modifications as canonical *U2*, suggesting that it likely constitutes a functional copy of *U2* rather than a pseudogene or the 5’UTR of *WDR74*. The presence of somatic mutations both in *U2* and *U2-2P* suggests that both copies might contribute to the tumorigenic process.

Finally, clinical analysis showed that the presence of the *U2* c.28C mutation is associated with a worse prognosis, including shorter TTFT in CLL, and shorter overall survival in prostate adenocarcinoma. In CLL, the association with a shorter TTFT was more evident in M-CLL than U-CLL. This difference could be due to the increased number of high risk driver mutations in U-CLL when compared to M-CLL^28,43^. Of note, in M-CLL, the prognostic value of *U2* mutations were similar to that of known mutated drivers of aggressive disease. Further studies are necessary to determine the association of the *U2* c.28C mutation with other driver alterations in CLL, as well as to determine its impact on additional tumor types for which the number of samples and/or available clinical data was scarce. Together, this study highlights the importance of exploring repetitive regions of the genome for a more comprehensive characterization of driver alterations in cancer, a process that will likely expand with the application of longer read sequencing technologies to cancer genomic analysis.

## Methods

### Armadillo

To identify somatic mutations in repetitive regions we developed a pipeline that we called Armadillo. The first step consists in the definition of repetitive regions of interest (RROI). For this aim, a BED file with regions of interest (i.e.: list of genes/exons) is provided, and then the reference sequence for each region is extracted and aligned to the reference genome using BLAT^44^. Regions mapped in multiple loci are marked as RROIs, and the thresholds to mark a region as repetitive are customizable. By default, thresholds are set at maximum 1 gap, >90% identity and difference in length <15%. If there are at least two hits matching the conditions, the region is considered as repetitive. For each RROI, the coordinates of all copies are saved into a text file as well as their sequence, with an extra 100 bp flanking each end to avoid the loss of borderline reads during the alignment step.

The next step takes a WGS BAM, and extracts all non-duplicated reads aligned to a RROI (defined as all loci to which the original region of interest could be mapped). Reads are then aligned using BWA-mem^5^ to a reference sequence for that RROI. This forces all reads from that RROI to be aligned to a single reference copy of that RROI. This procedure is performed for each RROI, and then all BAMs from a WGS sample are merged into one single BAM.

Once all reads from a RROI are aligned to the same single reference sequence, variant calling is performed. A pileup is generated from the tumor and non-tumor BAMs using SAMtools^45^. When a SNV is detected with at least 4 reads in the tumor and no more than 3 in the control, it is selected as a candidate variant. Reads containing the mutant base are re-aligned to the reference genome using BLAT, and any read having a perfect hit to the reference genome will be discarded. If there are at least 4 reads, analysis of the sequence context is performed. This step is aimed at detecting the presence of in *cis* variants, either due to changes in the reference sequence of the repeat or germline variants in that copy. It is expected that if a somatic mutation arises in a repeat containing a variant (or not containing it), all reads with the mutation should contain the in *cis* variant (or should not have it). In addition, this variant should also be observed in the non-tumor sample.

The posterior probability of the proportion of tumor mutant reads being greater than the control mutant reads was assessed with a Monte Carlo approximation. We used a beta-binomial distribution model to define the probability of detecting the variant allele frequency (VAF) with the mutant reads coverage (*y*) and the non-mutant context-supportive coverage (*n*) as shape parameters of the distribution.

1 $$p\left( {MAF{{{\mathrm{|}}}}y,n} \right) = Beta\left( {MAF{{{\mathrm{|}}}}y + 1,n + 1} \right)$$

The joint posterior for tumor and control for M simulations can be estimated as:

2 $$\begin{array}{l}p\left( {MAF_t,MAF_c{{{\mathrm{|}}}}y_t,n_t,y_c,n_c} \right) = p\left( {MAF_t{{{\mathrm{|}}}}y_t,n_t} \right)xp\left( {MAF_c{{{\mathrm{|}}}}y_c,n_c} \right) \\ = \left( {MAF_t^{\left( 1 \right)},MAF_c^{\left( 1 \right)}} \right),\left( {MAF_t^{\left( 2 \right)},MAF_c^{\left( 2 \right)}} \right),...,\left( {MAF_t^{\left( M \right)},MAF_c^{\left( M \right)}} \right)\end{array}$$

By following the Monte Carlo approximation, we estimated the posterior probability of the tumor *MAF* being greater than the control MAF as:

3 $$Pr\left[ {MAF_t \,>\, MAF_c} \right] \approx \frac{1}{{{{\mathrm{M}}}}}\mathop {\sum }\limits_{m = 1}^M {\Bbb I}\left( {MAF_t^{\left( m \right)} \,>\, MAF_c^{\left( m \right)}} \right)$$

i.e., we consider the probability of tumor *MAF* being greater than control *MAF* as the fraction of samples (simulations) where the *MAF*_*T*_ > *MAF*_*C*_. When it is lower than 0.95, we consider the variants as potentially germinal, so they are discarded. We determined that this procedure allowed us to correctly classify variants in >99% of cases, independently of the initial random seed.

This initial algorithm was originally run on the MCL and ICGC WGS samples. We manually reviewed all candidate variants reported by the pipeline. Reported mutations in this study are the ones considered as true positives during the reviewing process. We used the next criteria for keeping the variants: (i) at least four alternative allele supporting reads, (ii) high mean base quality in mutant reads, (iii) concordant cis-variants among all mutant reads, (iv) same cis-variants from mutant reads exist in the normal matched sample, (v) no strand or position bias. The program has low hardware requirements, and it is able to analyze a tumor-normal WGS pair each at 30× in less than 15 min using 30 CPUs and up to 14 GB of RAM for the 342 RROIs described.

### Samples and cell culture

Tumor and normal paired samples from 2240 cases from multiple ICGC projects (Supplementary Table 4), as well as 57 MCL samples^19^ were analyzed with Armadillo. We used all samples located at the collaboratory ICGC server, which includes 1,830 PCAWG donors, so when available, we used the PCAWG bam files. PRAD-CA samples were removed from the analysis due to an abnormal number of mutations in these samples. CLL cell line JVM3 (DSMZ) was grown in RPMI 1640, 10% FBS, 1% PSG and 1% non-essential amino acids. The study was approved by the Ethics Committee from Principado de Asturias and the Ethics Committee of the Hospital Clínic of Barcelona.

### Genome simulations

We used Bamsurgeon^14^ to generate simulated tumor genomes containing mutations in known positions to determine the sensitivity and specificity of Armadillo. We downsampled a 100× WGS from a healthy donor into two 30× BAM files, one was used as normal, and for the other we created 10 different BAMs introducing a total of 2,444 randomly selected positions from our RROIs to spike mutations in known sites. We did not introduce all mutations in a single simulated tumor genome to avoid the potential presence of several mutations in a small RROI, what could generate a bias due to soft-clipping or hard-clipping. The number of mutant reads assigned to these positions was based on the distribution of mutant reads observed for somatic mutations called by our standard pipeline for WGS analysis. Bamsurgeon was used with the next arguments: -z 150 --minmutreads 3 --mindepth 10 -s 1 -p 12 -d 0.8, and BWA was used to remap the reads selected by Bamsurgeon. After the generation of the tumor sample, positions with less than three mutant reads (*n* = 741) or those that did not align to the RROI (*n* = 6), were not considered for the analysis.

### Allelic discrimination assay

Allelic discrimination PCRs were performed on 822 CLL samples and 404 control patients (blood and bone marrow donors). rhAmp SNP assays from IDT were designed for targeting both *U2* and *U2-2P* (CD.GT.DXVP1237.1) to assess if the c.28C>T mutation exists in the gene or in the pseudogene. A *U2-2P* specific target was designed for *U2* – *U2-2P* discrimination (CD.GT.GYRH2013.1) when the previous test was positive. The reaction (5 μL reaction volume, 5 ng DNA) was performed according to the manufacturer’s indications. For tumor samples, a matched non-tumor sample from the same patient was analyzed only when the tumor sample tested positive, in order to discriminate between somatic and germline events.

### *U2-2P* expression analysis

To determine whether *U2-2P* was expressed, RNA-seq from total RNA from MCL and CLL samples was aligned to the hs37d5 (GRCh37 plus decoy) using STAR^46^. Only read pairs in which at least one read contained bases 108-111 of *U2* that discriminate between *U2* and *U2-2P*, were used for quantification and insert size analysis, as they can be unequivocally assign to each loci.

### Pseudouridylation analysis

The analysis of pseudouridylation in *U2* and *U2-2P* was performed using a primer extension modification method^47–49^ using specific oligonucleotides to distinguish *U2* from *U2-2P* (Supplementary Table 8). Briefly, total RNA from 15 × 10^6^ JVM3 cells was extracted with TRIzol and treated with CMC [N-cyclohexyl-N′-(2-morpholinoethyl) carbodiimidemetho-p-toluene sulfonate] (Sigma-Aldrich) for 20–30 min and then with pH 10.4 sodium carbonate buffer for 3–4 h at 37 °C. 6-FAM-labeled oligonucleotides for *U2* or *U2-2P* were used for primer extension (Supplementary Table 8) using AMV-RT (New England Biolabs) at 0.5 mM dNTP concentration. Extension products were ethanol precipitated and dissolved in formamide, mixed with GeneScandTM 400HD size standard and separated on an ABI 3130xl capillary electrophoresis instrument (Applied Biosystems).

### Spliceosome RNA immunoprecipitation

Spliceosome particles were immunoprecipitated with anti-SAP155 (D-221, MBL). Briefly, 20 × 10^6^ JVM3 cells were harvested, centrifuged and washed with PBS. Then, they were resuspended in 1 mL RIP buffer: 10 mM Tris/HCl pH 7.4, 100 mM NaCl, 2.5 mM MgCl_2_, 0.5% NP-40, 1× Complete protease inhibitor (Roche) and 40 U/mL RNAse inhibitor (Applied Biosystems). Cells were physically disrupted with a potter (15 strokes) after a 20-min incubation at 4 °C. Debris was pulled down by centrifugation at 4 °C (11,000 x g, 10 min). 150 μL slurry Dynabeads Protein G (ThermoFisher) were equilibrated with buffer C (25 mM NaCl, 25 mM Tris/HCl pH 7.4, 0.05% NP-40 and 2.5 mM MgCl_2_). The cell extract was then precleared with 50 μL equilibrated Dynabeads Protein G in rotation for 1 h. Then, 5% of sample was saved for total extract control for later experiments. Four μg anti-SAP155 (D221-3, MBL) and 4 μg anti-IgG (sc-2025, Santa Cruz) were incubated for 1 h in 50 μL equilibrated Dynabeads Protein G each. Finally, they were incubated rotating overnight with 50% cell extract each.

Beads were recovered with a magnet and washed twice with washing buffer A (1 M NaCl, 25 mM Tris/HCl pH 7.4, 0.05% NP-40, 2.5 mM MgCl_2_), twice with washing buffer B (400 mM NaCl, 25 mM Tris/HCl pH 7.4, 1% NP-40, 2.5 mM MgCl_2_) and twice with washing buffer C. The extract was resuspended in 50 μL PBS. Twenty μL were used for Western blot, while the remaining sample was used for RNA extraction with TRIzol.

Western blot was performed to validate the immunoprecipitation. We loaded 20 μL total extract, IgG IP and SAP155 (SF3B1) IP in an 8% polyacrylamide gel. Anti-SAP155 (sc-514655, Santa Cruz, diluted 1:1000) and anti-GAPDH (sc47724, Santa Cruz, diluted 1:1000) were used as primary antibodies. Goat anti-Mouse IgG (926-32210, LI-COR, diluted 1:10,000) was used for immunofluorescence detection of primary antibody. All blots were processed in parallel and derive from the same experiment.

RNA from immunoprecipitated spliceosome was purified by standard TRIzol-chloroform. One μL carrier glycogen (Invitrogen) was added during the precipitation step to improve the yield, since low RNA amounts were expected. RNA was dissolved in 10 μL DEPC water. RT-PCR of *U2-2P* was done in order to determine if it can be found in the spliceosome, similarly to *U2*. Reverse transcription (205314, Qiagen) was performed according to the manufacturer’s protocol. A 4-mer in *U2* allows the discrimination between *U2* and *U2-2P*. Therefore, PCR with 4-mer specific primers (Supplementary Table 8) were done for specific identification of *U2* and *U2-2P*. Also, a primer with a random 4-mer was used as negative control.

### Statistical analyses

*WDR74* expression, *U2* c.28C > T mutation expression and insert size distributions were compared with Wilcoxon signed-rank tests. Subsamples of BAM files (0.5%, ~100,000 reads) were used to extract insert sizes. Fisher’s exact test was used to compare PCAWG germline events with the healthy Spanish cohort (Supplementary Table 9). All test were two-sided unless otherwise specified.

### Clinical analyses

Primary end points studied were time to first treatment (TTFT) for the CLL cohort (Supplementary Table 9) and overall survival (OS) for the PRAD-UK cohort measured from time of sampling. Deaths previous to any treatment were considered competing events in TTFT analyses. Gray’s and log-rank tests were used to compare cumulative curves (TTFT) and Kaplan–Meier curves (OS), respectively. Multivariate models were modeled using the Fine-Gray (TTFT) and Cox (OS) regression models. For CLL, patients diagnosed with MCL were excluded.

### Reporting summary

Further information on research design is available in the Nature Research Reporting Summary linked to this article.

## Supplementary information

Supplementary Information

Supplementary Data

Reporting Summary Checklist

## Data Availability

All genomes and RNA-seq referenced can be accessed from the ICGC collaboratory server and EGA accessions EGAS00000000092 and EGAS00001004165. Somatic variants and samples in which they were found are listed in the Supplementary Tables. Materials and verification data that support the findings of this study are available from the corresponding author upon request.

## Code availability

Armadillo pipeline is available under MIT license through Github (https://github.com/xa-lab/Armadillo).
