# Supplementary material for: PanCancer analysis of somatic mutations in repetitive regions reveals recurrent mutations in snRNA U2
Source: NPJ Genom Med. 2022 Mar 14;7:19. doi: 10.1038/s41525-022-00292-2 (PMC8921233; doi:10.1038/s41525-022-00292-2)
Supplement: Supplementary file 1 — Supplementary Information [file 41525_2022_292_MOESM1_ESM.pdf]

Supplementary Material

PanCancer Analysis of Somatic Mutations in Repetitive Regions Reveals Recurrent Mutations in the Small Nuclear RNA U2

Pablo Bousquets-Muñoz[1], Ander Díaz-Navarro[1], Ferran Nadeu[2,3], Ana Sánchez-Pitiot[4], Sara López-Tamargo[1], Shimin Shuai[5], Milagros Balbín[4], Jose MC Tubio[6], Sílvia Beà[2,3,10], Jose I. Martin-Subero[2,3,7], Ana Gutiérrez-Fernández[1,3], Lincoln D. Stein[8,9], Elías Campo[2,3,10], Xose S Puente[1,3,*]

[1]Departamento de Bioquímica y Biología Molecular, Instituto Universitario de Oncología (IUOPA), Universidad de Oviedo, Oviedo, Spain

[2]Institut d'Investigacions Biomèdiques August Pi i Sunyer (IDIBAPS), Barcelona, Spain

[3]Centro de Investigación Biomédica en Red de Cáncer (CIBERONC), Spain

[4]Laboratorio de Oncología Molecular, Laboratorio de Medicina, Instituto Universitario de Oncología (IUOPA), Hospital Universitario Central de Asturias, Oviedo, Spain

[5]Department of Human Cell Biology and Genetics, School of Medicine, Southern University of Science and Technology, Shenzhen, China

[6]Genomes and Disease, Centre for Research in Molecular Medicine and Chronic Diseases (CIMUS), Universidade de Santiago de Compostela, Santiago de Compostela, Spain.

[7]Institució Catalana de Recerca i Estudis Avançats (ICREA), Barcelona, Spain

[8]Department of Molecular Genetics, University of Toronto, Toronto, Canada

[9]Computational Biology Program, Ontario Institute for Cancer Research, Toronto, Canada

[10]Hospital Clinic de Barcelona, Universitat de Barcelona

Correspondence:

Xose S Puente, Edificio Santiago Gascón, C/Fernando Bongera s/n, 3006 Oviedo, Spain. xspuente@uniovi.es, +34 985105027

Supplementary Figures

Supplementary Figure 1	Repetitive exons across gene sets encoding different RNA molecules
Supplementary Figure 2	Verification of U2 c.28C>T mutation in RNA-Seq data from CLL-ES samples
Supplementary Figure 3	GTEx quantification of *WDR74* junctions and location of *RNU2-2P*.
Supplementary Figure 4	Coverage at the *U2-2P* and *U2* locus from MCL cases with available RNA-Seq.
Supplementary Figure 5	Multiple sequence alignment of *U2* from different mammalian species and *U2-2P*.
Supplementary Figure 6	Clinical impact of the *U2* c.28C mutation in CLL patients.
Supplementary Figure 7	Oncoprint representation of driver alterations identified in CLL.

Uncropped gel and membrane figures

Uncropped gel from figure 4a (top)	RT PCR in immunoprecipitated samples
Uncropped membrane from figure 4a (bottom)	Western Blot of immunoprecipitated samples

Supplementary Tables

Supplementary Table 8	Oligonucleotides used for U2 and U2-2P
Supplementary Table 9	Characteristics of the cohorts

Supplementary Figures

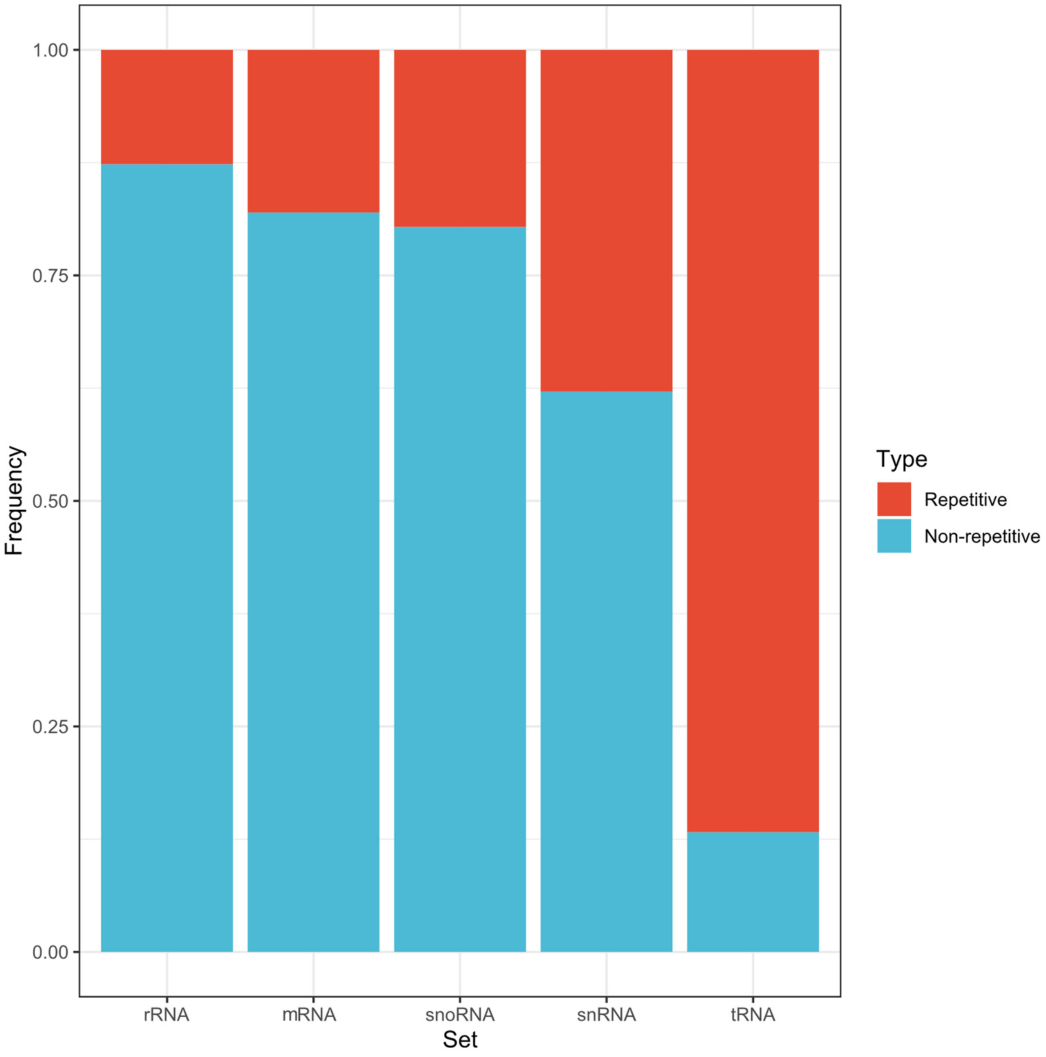

Supplementary Figure 1. Repetitive exons across gene sets encoding different RNA molecules. Percentage of exons from different gene types present in the human genome as unique or repetitive copies. Annotation was obtained from UCSC for rRNA and from BioMart for snRNA, snoRNA and mRNA. Repetitiveness was assessed by following thesame criteria used by Armadillo.

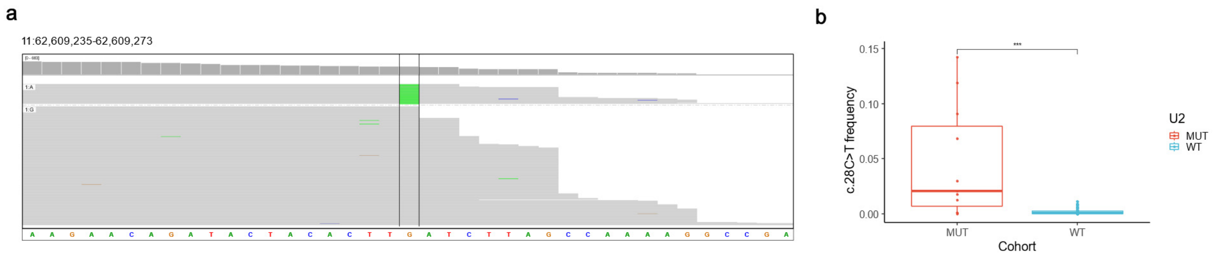

Supplementary Figure 2. Verification of U2 c.28C>T mutation in RNA-Seq data from CLL-ES samples. (**a**) Snapshot of RNA-seq reads from a MCL sample aligned to chr11:62,609,235-69,609,273 (GRCh37) showing the presence of c.28C>T mutated reads. (**b**) Frequency of reads supporting the c.28C>T mutation in RNA-seq data from CLL and MCL samples determined as mutated by rhAmp (N=13) versus wild type samples (N=95). Boxplots elements represent: center line = median, upper and lower hinges = 25 and 75% percentiles, upper and lower whisker = mean ± 1.5*IQR.***, P=5.7·10-5, Wilcoxon test.

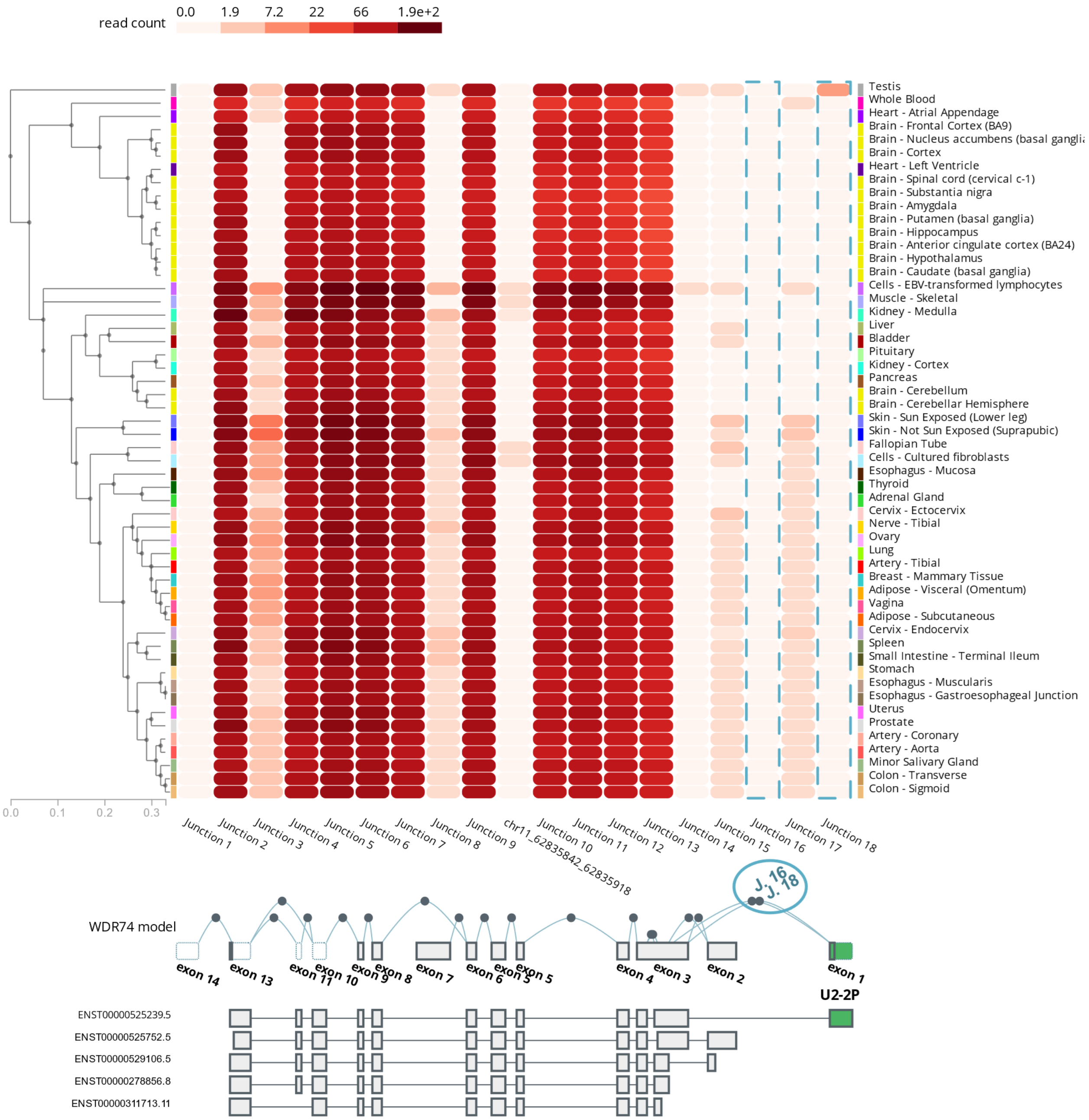

Supplementary Figure 3. Quantification of exon expression and junction usage for WDR74 obtained from GTEx. Below is a model for WDR74, showing in green the location of RNU2-2P, which is specifically expressed in testis and spliced through two junctions (junction 16 or 18, highlighted) to exon 3 of the model.

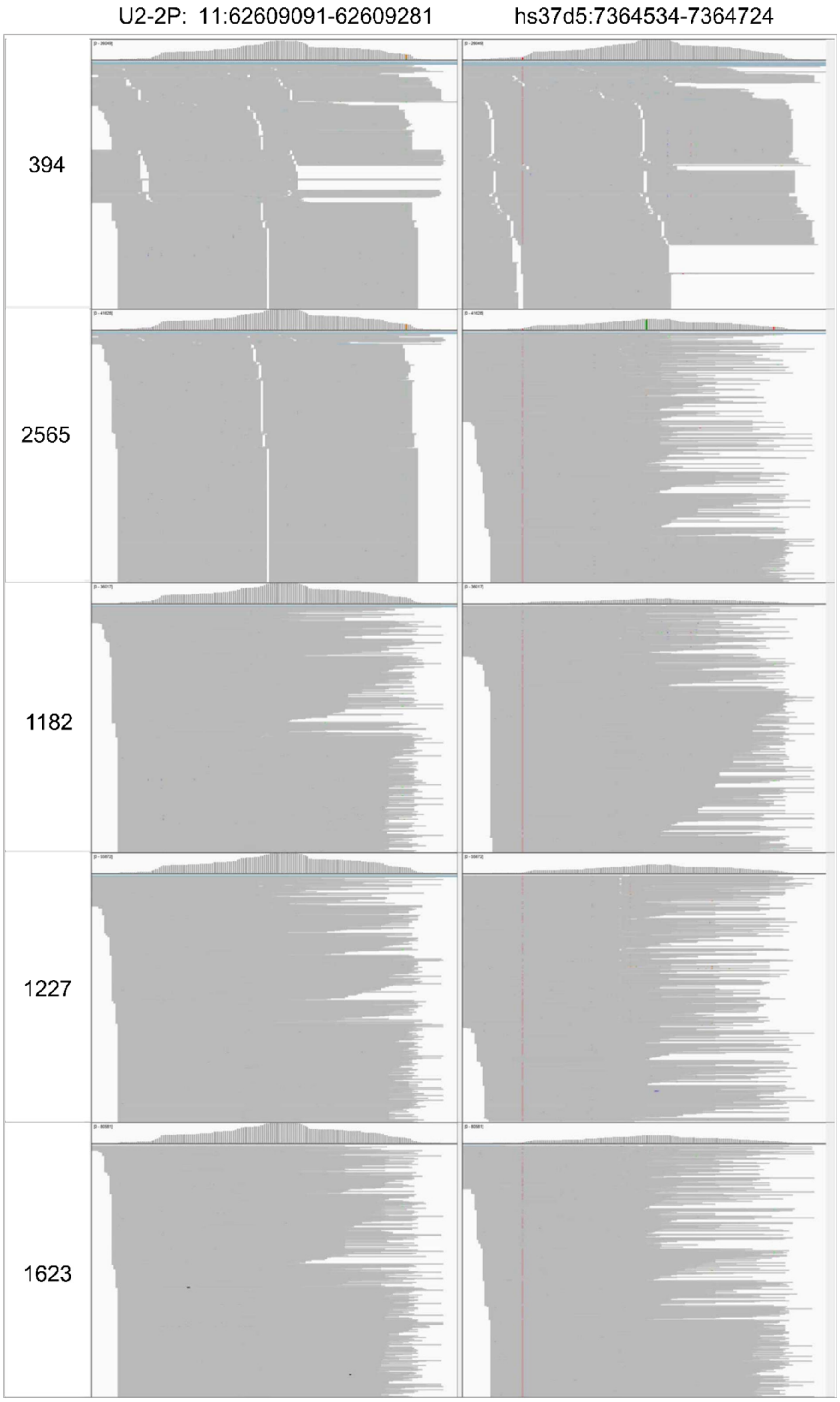

Supplementary Figure 4. Representative snapshots of RNA-seq data at the U2 and U2-2P loci. RNA-Seq was aligned with the hs37d5 reference genome, which contains one U2 copy as well as the U2-2P copy at chromosome 11. Read pairs unequivocally belonging to U2 or U2-2P based on the differences in sequence were plotted. Note that the reference decoy sequencehas a change with respect to canonical U2, resulting in the detection of a variant in all U2-derived reads aligned to the decoy sequence, while sample 394 and 2565 have the c.28C>T mutation in U2-2P.

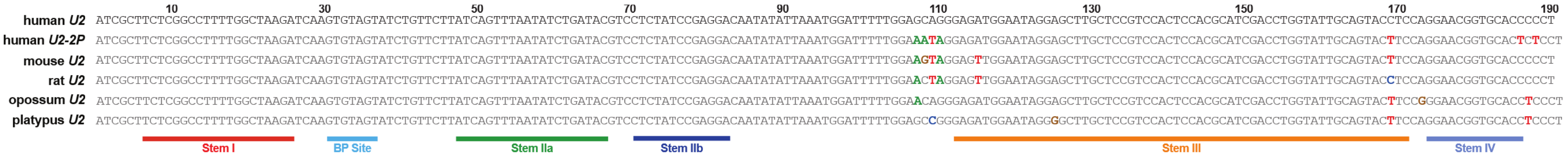

Supplementary Figure 5. Multiple sequence alignment of U2 from different mammalian species and U2-2P. Differential bases are shown in different colours, and the location of secondary structures is indicated in the bottom.

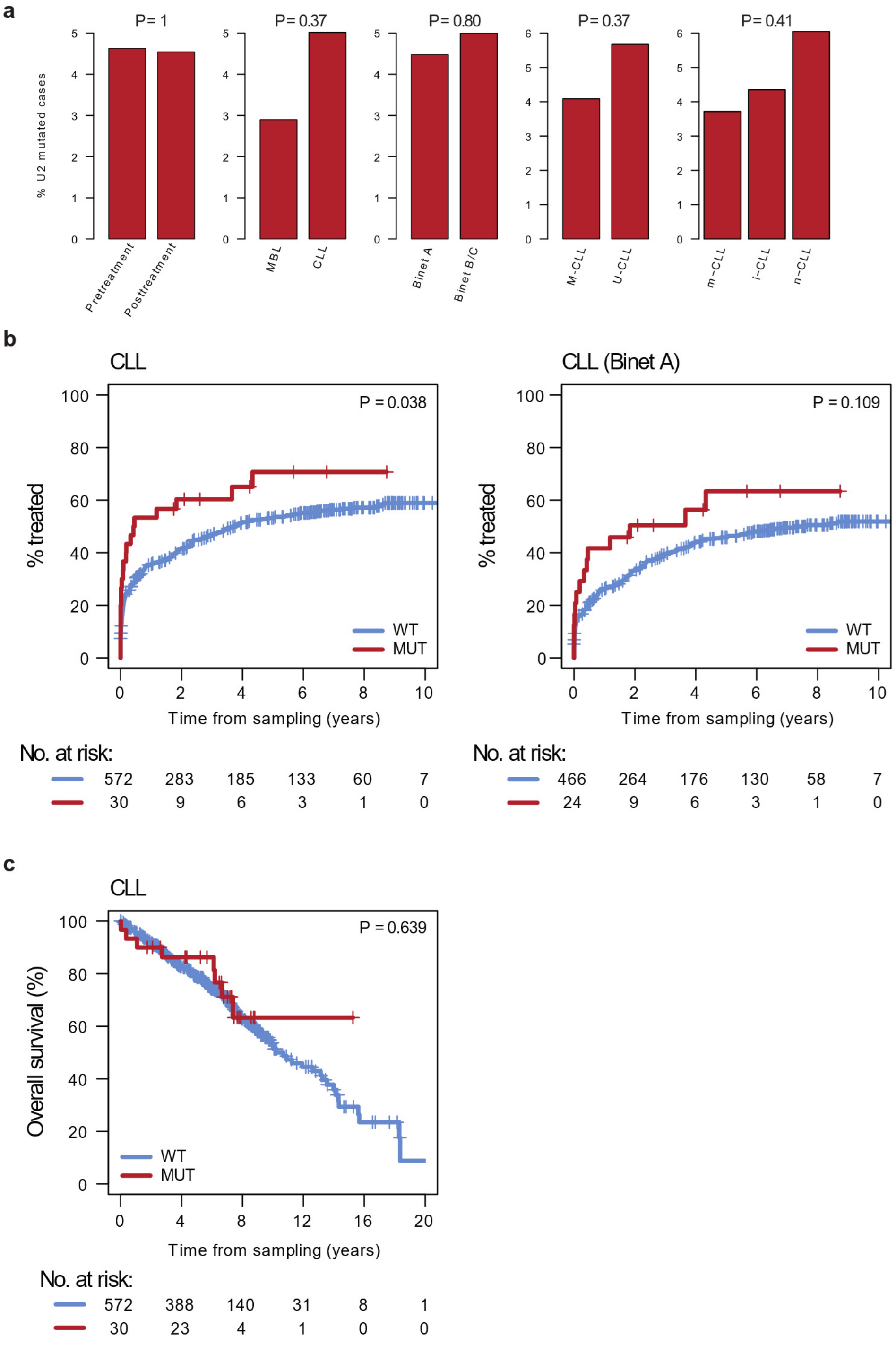

Supplementary Figure 6. Clinical impact of the U2 c.28C mutation in CLL patients. (**a**) Distribution of U2 c.28C mutated cases across typical CLL covariates (treatment, diagnosis, Binet stage, IGHV mutational status or epigenetic subgroup). (**b**) Effect of the U2 c.28C mutation on TTFT in all CLL cases (left) or only in Binet stage A patients (right). (**c**) Kaplan-Meier analysis showing overall survival in CLL patients according to the presence of the U2 c.28C mutation.

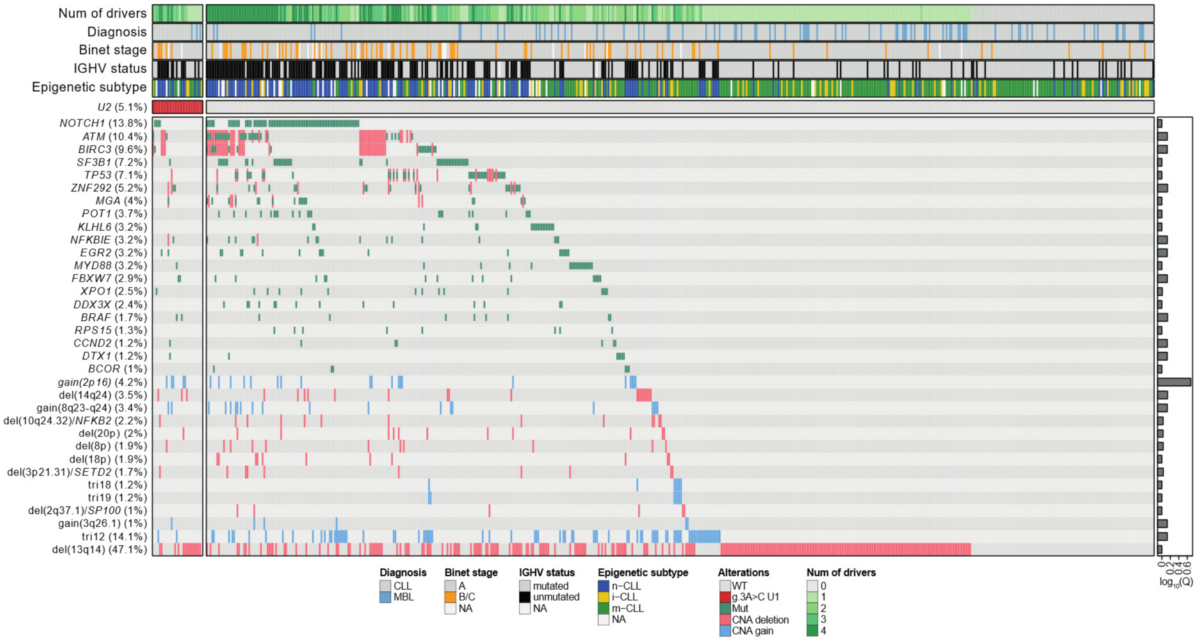

Supplementary Figure 7. Oncoprint representation of driver alterations identified in CLL. Drivers are depicted in rows and cases in columns. Log_{10}(Q-value) of co-ocurrence of U2 mutations with other driver alterations is shown on the right.

Uncropped gel and membrane figures

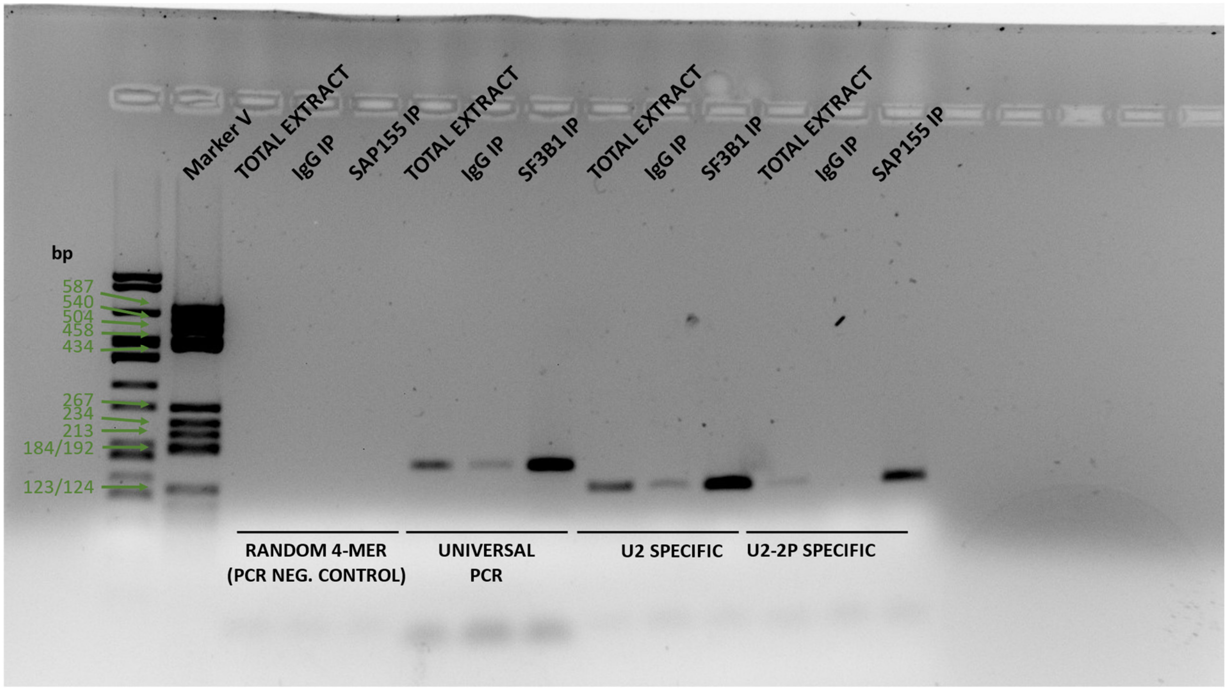

Uncropped gel from figure 4a (top). RT PCR in immunoprecipitated samples.

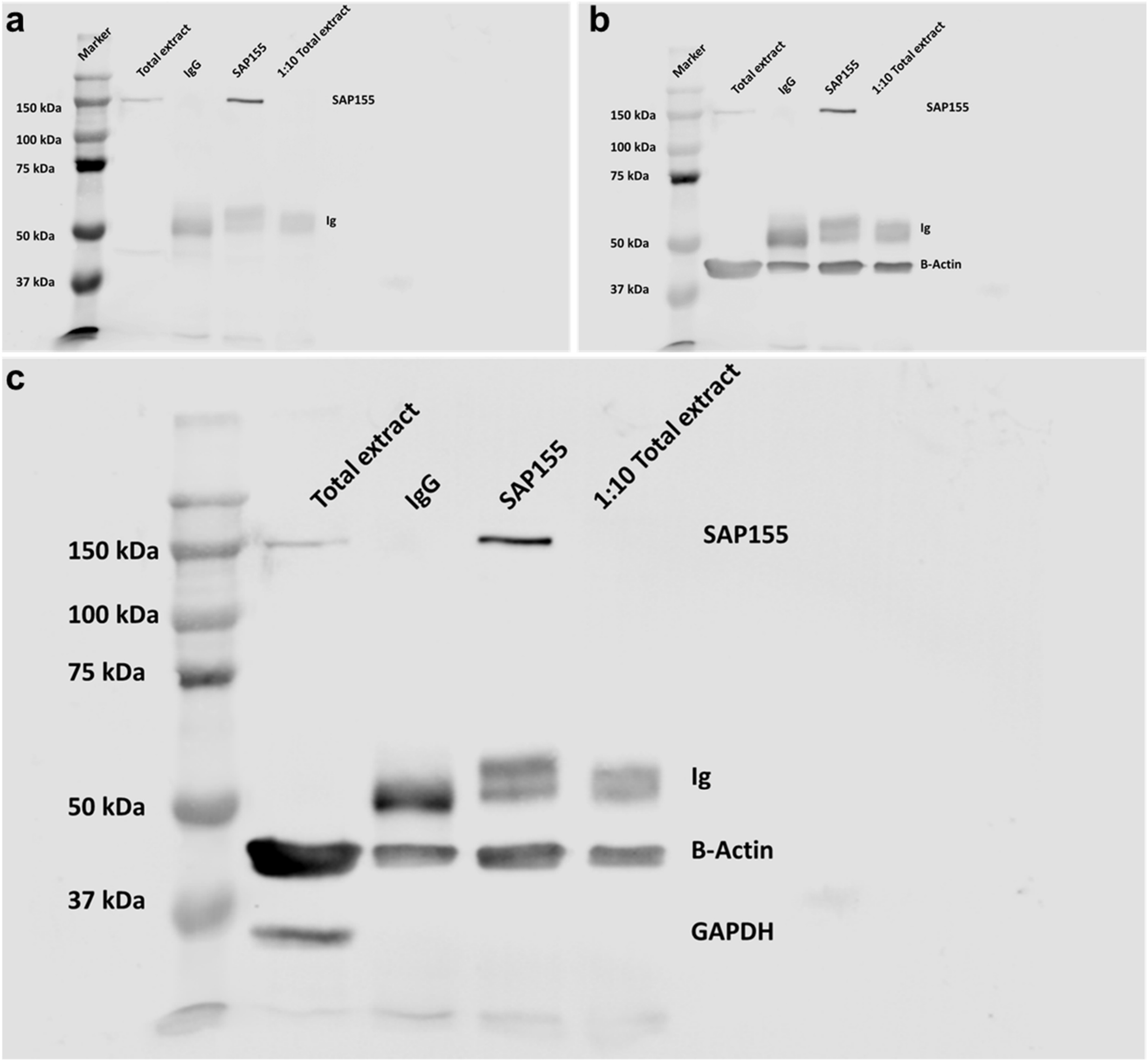

Uncropped membrane from figure 4a (bottom). Western Blot of immunoprecipitated samples. The membrane was incubated sequentially with anti-SAP155 (sc-514655, 1:1000) and Goat anti-mouse (926-32210, 1:10,000) (**a)**; then anti-βactin (AC-15, Sigma, 1:5,000), and Goat anti-mouse (926-32210, 1:10,000) (**b**); and finally with anti-GAPDH (sc-47724, 1:1,000) and Goat anti-mouse (926-32210, 1:10,000) (**c**). Marker is Precision Plus Protein™ Dual Color Standards (1610394, Bio-Rad).

Supplementary Tables

Name	Strand	Comment	Experiment	Seq (5'-3')
U2/U2-2P_RT_Fwd	Fwd	Anneal both at U2 and U2-2P	RT-PCR	ATCGCTTCTCGGCCTTTTGG
U2/U2-2P_Rev	Rev	Anneal both at U2 and U2-2P	RT-PCR	TACCAGGTCGATGCGTGGA
U2-2P_RT_Rev	Rev	U2-2P specific	RT-PCR	CTCCTATTCCATCTCCTATT
U2_RT_Rev	Rev	U2 specific	RT-PCR	CTCCTATTCCATCTCCCTGC
MOCK_RT_Rev	Rev	Negative control	RT-PCR	CTCCTATTCCATCTCCAGCA
U2-2P_FAM	Rev	U2-2P specific	Pseudouridines	/56-FAM/CTCCTATTCCATCTCCTATT
U2_FAM	Rev	U2 specific	Pseudouridines	/56-FAM/CTCCTATTCCATCTCCCTGC

Supplementary Table 8. Oligonucleotides used for U2 and U2-2P amplification

	CLL cohort (n=823)	Control cohort (n=401)
**Age at sampling (mean, range)**	67.6 (19-91)	49 (n=374)
**Sampling date**		
Pretreatment	756	
Posttreatment	67	
**Patients treated during follow-up (pretreatment cohort)**	332/756 (44%)	
**Sex**		
Male	477	188
Female	330	212
NA	16	1
**Binet stage**		
A	664	
B	94	
C	31	
NA	34	
**IGHV mutational status**		
M-CLL	497	
U-CLL	316	
NA	10	
**Epigenetic subtype**		
m-CLL	366	
i-CLL	96	
n-CLL	234	
NA	127	

Supplementary Table 9. Characteristics of the cohorts
