# Supplementary material for: PanCancer analysis of somatic mutations in repetitive regions reveals recurrent mutations in snRNA U2
Source: NPJ Genom Med. 2022 Mar 14;7:19. doi: 10.1038/s41525-022-00292-2 (PMC8921233; doi:10.1038/s41525-022-00292-2)
Supplement: Supplementary file 3 — Reporting Summary Checklist [file 41525_2022_292_MOESM3_ESM.pdf]

nature portfolio

Corresponding author(s): Xose S Puente

Last updated by author(s): Jan 14, 2022

Reporting Summary

Nature Portfolio wishes to improve the reproducibility of the work that we publish. This form provides structure for consistency and transparency in reporting. For further information on Nature Portfolio policies, see our Editorial Policies and the Editorial Policy Checklist.

Statistics

For all statistical analyses, confirm that the following items are present in the figure legend, table legend, main text, or Methods section.

n/a	Confirmed	
☐	☒	The exact sample size (*n*) for each experimental group/condition, given as a discrete number and unit of measurement
☒	☐	A statement on whether measurements were taken from distinct samples or whether the same sample was measured repeatedly
☐	☒	The statistical test(s) used AND whether they are one- or two-sided<br>*Only common tests should be described solely by name; describe more complex techniques in the Methods section.*
☐	☒	A description of all covariates tested
☐	☒	A description of any assumptions or corrections, such as tests of normality and adjustment for multiple comparisons
☐	☒	A full description of the statistical parameters including central tendency (e.g. means) or other basic estimates (e.g. regression coefficient) AND variation (e.g. standard deviation) or associated estimates of uncertainty (e.g. confidence intervals)
☐	☒	For null hypothesis testing, the test statistic (e.g. *F*, *t*, *r*) with confidence intervals, effect sizes, degrees of freedom and *P* value noted<br>*Give P values as exact values whenever suitable.*
☐	☒	For Bayesian analysis, information on the choice of priors and Markov chain Monte Carlo settings
☒	☐	For hierarchical and complex designs, identification of the appropriate level for tests and full reporting of outcomes
☒	☐	Estimates of effect sizes (e.g. Cohen's *d*, Pearson's *r*), indicating how they were calculated

Our web collection on statistics for biologists contains articles on many of the points above.

Software and code

Policy information about availability of computer code

Data collection	The ICGC's score-client was used for data collection (https://github.com/overture-stack/score/releases)
Data analysis	Data analysis was performed using custom code called Armadillo which is available at https://github.com/xa-lab/Armadillo<br>The following published code was used:<br>BWA (built with v.0.7.17)<br>Samtools (built with v.1.9)<br>gfClient (built with v.35)<br>Python3 (built with v.3.7)<br>Statistics and figures were generated with R version 3.6.3 (2020-02-29)

For manuscripts utilizing custom algorithms or software that are central to the research but not yet described in published literature, software must be made available to editors and reviewers. We strongly encourage code deposition in a community repository (e.g. GitHub). See the Nature Portfolio guidelines for submitting code & software for further information.

Data

Policy information about availability of data

All manuscripts must include a data availability statement. This statement should provide the following information, where applicable:

- Accession codes, unique identifiers, or web links for publicly available datasets
- A description of any restrictions on data availability
- For clinical datasets or third party data, please ensure that the statement adheres to our policy

All genomes and RNA-seq referenced can be accessed from the ICGC collaboratory server and EGA accessions EGAS00000000092 and EGAS00001004165. Somatic

variants and samples in which they were found are listed in the Supplementary Tables. Materials and verification data that support the findings of this study are available from the corresponding author upon request.

Field-specific reporting

Please select the one below that is the best fit for your research. If you are not sure, read the appropriate sections before making your selection.

☒ Life sciences ☐ Behavioural & social sciences ☐ Ecological, evolutionary & environmental sciences

For a reference copy of the document with all sections, see nature.com/documents/nr-reporting-summary-flat.pdf

Life sciences study design

All studies must disclose on these points even when the disclosure is negative.

Sample size	Sample size was limited by the number of cases available at the ICGC PCAWG for the discovery phase, and it was extended to 831 CLL samples for verification which were the largest number of samples available and enough to confirm the U2 mutation and explore its clinical impact.
Data exclusions	Prostate adenocarcinoma samples from the PRAD-CA project in the PCAWG data were removed from the analysis due to quality control issues as an abnormal number of mutations were detected only in this series.
Replication	We have confirmed the reproducibility of the findings by performing orthogonal validation in the same samples, and all attempts were successful.
Randomization	Patients were classified according to the main driver mutations present, mutations in U2, and whether they had mutations in IGHV genes, as usual for CLL analysis.
Blinding	Blinding was not relevant for the study.

Reporting for specific materials, systems and methods

We require information from authors about some types of materials, experimental systems and methods used in many studies. Here, indicate whether each material, system or method listed is relevant to your study. If you are not sure if a list item applies to your research, read the appropriate section before selecting a response.

Materials & experimental systems

n/a	Involved in the study	
☐	☒	Antibodies
☐	☒	Eukaryotic cell lines
☒	☐	Palaeontology and archaeology
☒	☐	Animals and other organisms
☒	☐	Human research participants
☐	☒	Clinical data
☒	☐	Dual use research of concern

Methods

n/a	Involved in the study	
☒	☐	ChIP-seq
☒	☐	Flow cytometry
☒	☐	MRI-based neuroimaging

Antibodies

Antibodies used	mouse anti-SAP155: MBL (D-221-3, lot #024, clone 16). RIP and Western Blot.<br>mouse anti-SAP155: Santa Cruz (sc-514655, lot #C3017).Western Blot.<br>mouse anti-GAPDH: Santa Cruz (sc-47724, lot #0411). Western Blot<br>anti-normal mouse IgG: Santa Cruz (sc-2025, lot #G0921). RIP Negative control.<br>goat anti-mouse: LI-COR (926-32210, lot #C808 16-15). Secondary antibody, Western Blot
Validation	- SAP155 (D-221-3): Kotake, Y., Sagane, K., Owa, T. et al. Splicing factor SF3b as a target of the antitumor natural product pladienolide. Nat Chem Biol 3, 570–575 (2007). https://doi.org/10.1038/nchembio.2007.16<br>- SAP155 (sc-514655): Jin, L., Chen, Y., Crossman, D.K. et al. STRAP regulates alternative splicing fidelity during lineage commitment of mouse embryonic stem cells. Nat Commun 11, 5941 (2020). https://doi.org/10.1038/s41467-020-19698-6<br>- anti-GAPDH: Validated by manufacturers at https://www.scbt.com/es/p/gapdh-antibody-0411<br>- anti-normal mouse IgG: Bejjani F, Tolza C, Jariel-Encontre I et al. Fra-1 regulates its target genes via binding to remote enhancers without exerting major control on chromatin architecture in triple negative breast cancers. Nucleic Acids Res (2021). https://doi.org/10.1093/nar/gkab053<br>- goat anti-mouse (926-32210): Wilson, C., Chen, P.J., Miao, Z. et al. Programmable m6A modification of cellular RNAs with a Cas13-directed methyltransferase. Nat Biotechnol 38, 1431–1440 (2020). https://doi.org/10.1038/s41587-020-0572-6

Eukaryotic cell lines

Policy information about cell lines

Cell line source(s)	CLL cell line was obtained from DSMZ (https://www.dsmz.de/catalogues/catalogue-human-andanimal-cell-lines.html).
Authentication	The authenticitv of the cell lines was tested with the AmpFLSTR Identifiler Plus PC Amplification Kit.
Mycoplasma contamination	Cells tested negative for mycoplasma
Commonly misidentified lines (See ICLAC register)	No misidentified lines were used

Clinical data

Policy information about clinical studies

All manuscripts should comply with the ICMJE guidelines for publication of clinical research and a completed CONSORT checklist must be included with all submissions.

Clinical trial registration	*Provide the trial registration number from ClinicalTrials.gov or an equivalent agency.*
Study protocol	*Note where the full trial protocol can be accessed OR if not available, explain why.*
Data collection	*Describe the settings and locales of data collection, noting the time periods of recruitment and data collection.*
Outcomes	*Describe how you pre-defined primary and secondary outcome measures and how you assessed these measures.*
